# Phlorizin ameliorates obesity-associated endotoxemia and insulin resistance in high-fat diet-fed mice by targeting the gut microbiota and intestinal barrier integrity

**DOI:** 10.1080/19490976.2020.1842990

**Published:** 2020-11-23

**Authors:** Xiao-Yu Zhang, Jiang Chen, Kang Yi, Ling Peng, Jie Xie, Xun Gou, Tong Peng, Lin Tang

**Affiliations:** a College of Life Sciences, Sichuan Normal University, Chengdu, P.R. China; b College of Life Sciences, Sichuan University, Chengdu, P.R. China; c Chengdu Institute of Biology, Chinese Academy of Sciences, Chengdu, P.R. China; d R&D Department, Keystonecare Technology (Chengdu) Co., Ltd, Chengdu, P.R. China

**Keywords:** Phlorizin (PHZ), obesity, gut microbiota, short-chain fatty acids (SCFAs), glucagon-like peptide 2 (GLP-2), barrier integrity

## Abstract

Phlorizin (PHZ) is one of phytonutrients in apples that contributes to the health-promoting effect implicated by the saying, ‘an apple a day keeps the doctor away’. PHZ was firstly identified as a competitive inhibitor of sodium-glucose co-transporters-2 (SGLT2); however, its low bioavailability makes it hard to fully explain its pharmacological mechanisms. This study aimed to investigate the ameliorating effect of PHZ on high-fat diet (HFD)-induced obesity via modulating the “gut microbiota-barrier axis”. Firstly, C57BL/6 J mice were fed a normal chow diet (NCD) or HFD coadministered with or without PHZ for 12 weeks. Our results showed that PHZ supplementation significantly reduced HFD-induced body weight gain (*P < *.001), alleviated metabolic disorders (MDs) like insulin resistance (*P < *.001) and elevation of serum lipopolysaccharides (LPS) (*P < *.001), attenuated HFD-induced gut microbiota alterations, enhanced short-chain fatty acids (SCFAs) production (*P < *.001), and inhibited fecal LPS production (*P < *.001). To investigate the role of the fecal microbiota in the observed beneficial effects, a fecal microbiota transplantation (FMT) experiment was performed by transplanting the feces of the four groups of mice (as donor mice) daily collected from the fourth week to a new batch of acclimatized HFD-fed mice. Our results confirmed that feeding the gut contents of the PHZ-modulated mice could attenuate HFD-induced MDs, accompanied by enhanced glucagon-like peptide 2 (GLP-2) secretion (*P < *.001) and restoration of HFD-induced damage in the gut epithelial barrier. This study has provided evidence that the “gut microbiota-barrier axis” was an alternative target for the anti-obesity effect of PHZ. This work has also provided an explanation for the high efficacy of PHZ despite the low bioavailability, and PHZ holds great potential to be developed as a functional food ingredient.

## Introduction

The well-known expression, ‘an apple a day keeps the doctor away’, was originated from an old English saying, ‘ate an apfel avore gwain to bed makes the doctor beg his bread’,^1^ implicating the health-promoting effects of eating apples. The health benefits of apples are partly attributed to the rich phytonutrients, such as phlorizin (PHZ, also known as phloridzin). Phytonutrients are normally acquired by eating healthy diets, and they are associated with disease interventions.^2–4^

PHZ is a glucoside of phloretin (PHT) belonging to the chemical class of dihydrochalcone. It was firstly extracted by French chemists from the root bark of apple (*Malus domestica*),^5^ with a much higher content found in peels (12 to 418 mg/kg) than in the pulp of different varieties (4 and 20 mg/kg).^6^ Our previous work found that *Docynia indica* could be an alternative source of PHZ, containing up to 25% of PHZ in dried leaves.^7^ For over 160 years, PHZ has been used as a pharmaceutical tool in physiological research, as it is a competitive inhibitor of sodium-glucose co-transporter 2 (SGLT2), which is responsible for approximately 90% of glucose reabsorption.^6^ In other words, PHZ holds great potential for improving hyperglycemia (one of the symptoms of metabolic disorders (MDs), like obesity and type 2 diabetes mellitus (T2DM)), by directly decreasing the blood glucose concentration rather than targeting insulin resistance and impaired insulin secretion.^8,9^ However, despite the fact that SGLT2 is regarded as a new class of therapeutic targets for managing MDs,^8,9^ currently only few PHZ-based products are designed and available for such purpose mainly due to the extremely low bioavailability of PHZ.^9,10^ Indeed, a large amount of ingested but unabsorbed PHZ will reach the gastrointestinal tract, and some of which will be hydrolyzed to PHT.^11^

Gastrointestinal disorders induced by high-fat diet (HFD) are associated with MDs, including abnormal blood glucose metabolism, elevated triglycerides, and decreased high-density lipoproteins, contributing to the development of obesity, T2DM, and cardiovascular disease.^12,13^ The continuous increase in the prevalence of obesity has made it the most prevailing nutritional disorder and the biggest public health challenge.^14^ In 2000, the World Health Organization has already declared obesity as a global epidemic.^15^ Currently, more than 1.9 billion people are obese and are at increased risk for developing MDs, such as obesity, T2DM, cardiovascular and liver diseases.^16^ Therefore, multifaceted therapeutic approaches are desperately needed to terminate the cascade from neonatal adiposity/high birth weight to childhood excessive weight gain/adult obesity with comorbidities.^14^ Recently, abundant experimental and clinical studies have provided evidence supporting the possibility of gut microbiota as a new target for controlling the obesity epidemic.^17–20^

Numerous studies have reported that administering HFD could alter the gut microbiota and the gut environment in various ways, e.g., reducing the gut bacterial diversity, shifting the ratio of gut *Firmicutes* to *Bacteroides*, suppressing some favorable bacteria (e.g., *Bifidobacterium*) while stimulating the potentially harmful ones (e.g., *Desulfovibrionaceae*), and changing the gut levels of short-chain fatty acids (SCFAs) and lipopolysaccharide (LPS).^21,22^ These alterations are consistently associated with adipose tissue growth, systemic inflammation, and metabolic comorbidities in humans and animals.^23^ Endotoxemia is an important clinical indicator of MDs like obesity.^24^ It is characterized by an elevated level of plasma LPS, often resulted from HFD-induced microbial gut dysbiosis.^19^ The intestinal LPS can damage the gut barrier integrity,^25^ leading to the leakage of LPS to the systemic circulation, inducing chronic inflammation, and contributing to metabolic endotoxemia and obesity. Thus, both the gut microbiota and the physical barrier of the gastrointestinal tract are considered as therapeutic targets for MDs.^26–31^

The role of PHZ as a competitive inhibitor of SGLT2 might potentially help alleviate hyperglycemia and thus improve MDs-associated conditions; however, providing the extremely low bioavailability of PHZ, it is hard to completely explain the therapeutic efficacy. Based on the facts that a large portion of PHZ would enter the gut after ingestion and that the gut microbiota could play a major role in protecting the gut barrier integrity and inhibiting LPS leakage into the systemic circulation, this study hypothesized that the gut microbiota was an alternative target for PHZ to alleviate MDs-associated conditions. The study aimed to first show the anti-obesity effect and therapeutic mechanisms of PHZ administration in a murine HFD model, followed by confirming the role of the gut microbiota in the beneficial effect by using fecal microbiota transplantation (FMT).

## Results

### PHZ inhibited HFD-induced obesity-related symptoms

**Figure 1.**
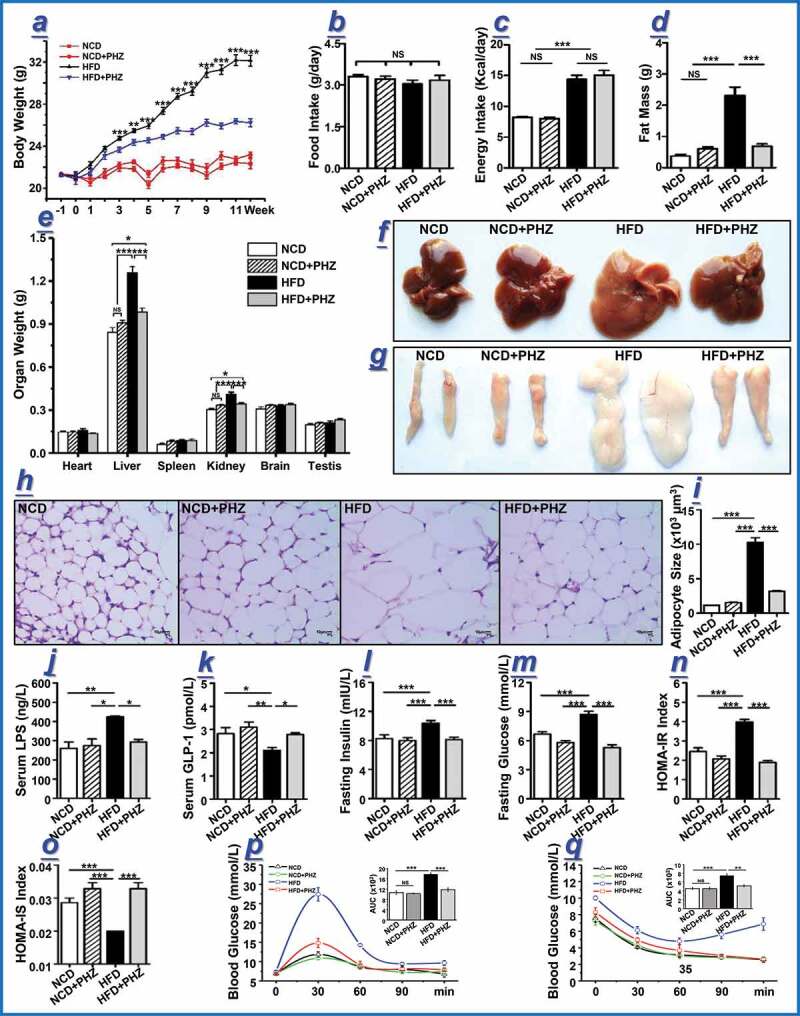
Phlorizin (PHZ) attenuated high-fat diet (HFD)-induced obesity. (a) Changes in the body weight of mice over 12 weeks. At week 12, multiple obesity-related parameters were recorded for the four groups of mice receiving normal chow diet (***NCD***), NCD with PHZ (***NCD+PHZ), HFD***, and HFD with PHZ (***HFD+PHZ***), respectively. The parameters included: (b) food intake; (c) energy intake; (d) fat mass; (e) weights of different organs; morphological observations of the (f) liver and (g) epididymis fat; (h) hematoxylin and eosin staining of epididymis fat; (i) adipocyte size of epididymis fat; levels of serum (j) lipopolysaccharide (LPS) and (k) glucagon-like peptide-1 (GLP-1); (l) fasting blood insulin; (m) fasting glucose; (n) homeostasis model assessment (HOMA)-insulin resistance (IR) index; (o) HOMA-insulin sensitivity (IS) index; (p) oral glucose tolerance test; (q) insulin tolerance test. Data are expressed as mean ± standard deviation. One-way ANOVA was used to analyze statistical differences; NS for *P* > .05, *P < .05, **P < .01, and ***P < .001

C57BL/6 J mice fed a NCD or a HFD were coadministered with or without PHZ (80 mg/kg) for 12 weeks. The results showed that PHZ coadministration effectively suppressed HFD-induced weight gain, resulting in a significantly lower body weight from the 3^rd^ week (*P* < .001; Figure 1a). However, no significant difference was found in the food intake (Figure 1b) or the energy intake (Figure 1c) between the NCD and NCD+PHZ groups, or between the HFD and HFD+PHZ groups (*P > *.05 in all cases), indicating that the anti-obesity effect resulted from PHZ coadministration was not due to reduction in food consumption or energy extraction. This was further confirmed by the decreases in fat accumulation (Figure 1d) and changes in the weights of different organs (Figure 1e). Morphological observations indicated that PHZ significantly inhibited HFD-induced fat accumulation in the liver (Figure 1f), which might effectively reduce further development of complicating diseases, such as fatty liver. On the other hand, PHZ significantly inhibited HFD-induced fat accumulation (Figure 1g) and fat cell expansion (Figure 1h,i, *P < *.001).

As a component of the Gram-negative bacterial cell wall,^32^ LPS is regarded as a triggering factor that promotes the onset and progression of systemic inflammation and related MDs by multiple pathways.^20^ Moreover, HFD-induced LPS elevation results in endotoxemia.^17^ Similarly, this study found a significantly elevated serum LPS level in HFD-fed mice (*P* < .01), and PHZ supplementation significantly inhibited the increase in serum LPS (*P* < .05; Figure 1j). Long-term systemic inflammation has also been proven to trigger glucose tolerance and insulin resistance,^33^ which are strongly associated with the onset and progression of obesity. As one of the most important gut hormones, glucagon-like peptide 1 (GLP-1) stimulates glucose-dependent insulin secretion and consequently improves metabolic syndromes, such as obesity and T2DM.^34^ In the current study, the serum GLP-1 level was significantly reduced by HFD (*P* < .05 compared with the NCD group; *P* < .01 compared with the NCD+PHZ group); however, such effect was significantly inhibited by PHZ supplementation (*P* < .05; Figure 1k). As a result, the elevated levels of fasting blood insulin (Figure 1l), glucose (Figure 1m), and HOMA-IR (Figure 1n) induced by HFD were significantly prevented (*P* < .001 in all cases). On the other hand, the HOMA-IS level was significantly suppressed by HFD, but such effect was also prevented by PHZ supplementation (*P* < .001; Figure 1o). Besides, the mice in the HFD+PHZ group showed significantly lower area under the curve (AUC) values in the oral glucose tolerance test (OGTT; Figure 1p) and insulin tolerance test (ITT; Figure 1q) compared with those in the HFD group (*P* < .001 and *P* < .01, respectively).

### PHZ attenuated HFD-induced gut microbial and metabolic dysbiosis

**Figure 2.**
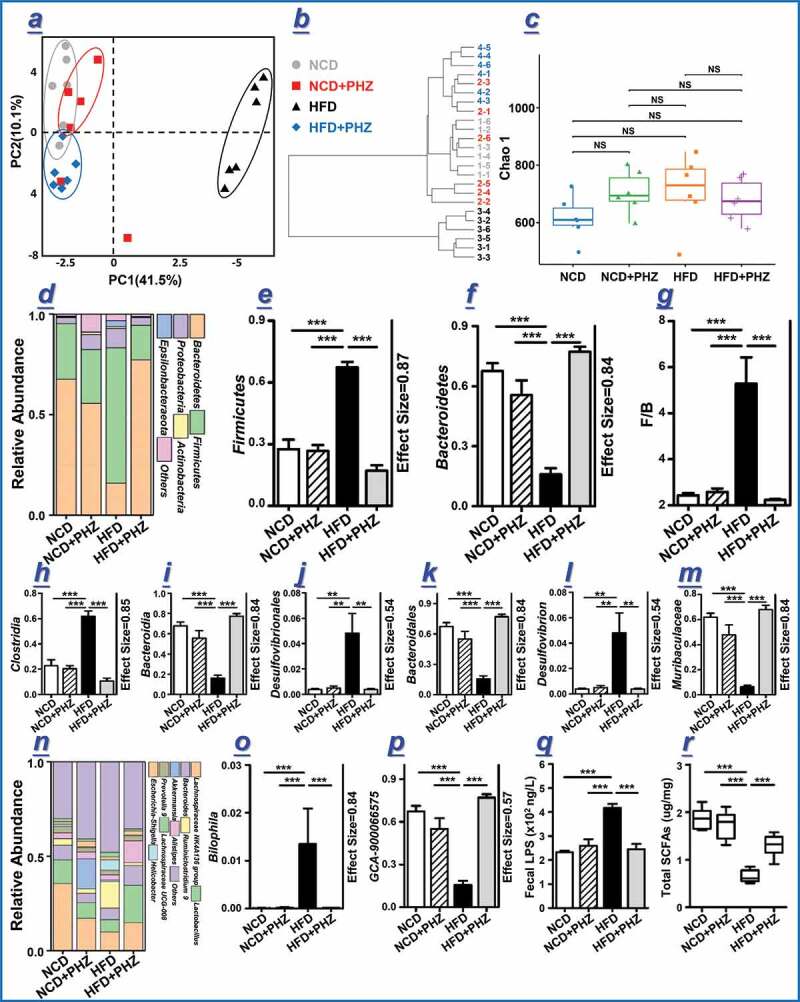
Phlorizin (PHZ) attenuated high-fat diet (HFD)-induced microbial and metabolic dybiosis. (a) Principal components analysis (PCA) score plot and (b) hierarchical clustering of fecal microbiota of the four groups of mice receiving normal chow diet (***NCD***), NCD with PHZ (***NCD+PHZ), HFD***, and HFD with PHZ (***HFD+PHZ***), respectively. (c) Chao 1 index representing the α-diversity of the gut microbiota. (d) Phylum-level distribution of fecal microbiota; (e, f) relative abundance of the phyla *Firmicutes* and *Bacteroidetes*; (g) ratio between the relative abundance of *Firmicutes* and *Bacteroidetes*; (h-m, o-p) relative abundance of identified differential abundant bacterial groups at different taxonomic levels; (n) Genus-level distribution of fecal microbiota; (q) fecal lipopolysaccharide (LPS); (r) total short-chain fatty acids (SCFAs). Data are expressed as mean ± standard deviation. One-way ANOVA was used to analyze statistical differences; NS for *P* > .05, **P < .01 and ***P < .001

Previous works have demonstrated a critical role of PHZ in preventing and treating MDs by directly decreasing the blood glucose level via competitive inhibition of SGLT2;^35,36^ however, the extremely low bioavailability of PHZ casted doubt on whether this was the only mechanism behind the therapeutic effect.^10^ Thus, the current work hypothesized that the gut microbiota was an alternative target responsible for the observed beneficial effects of PHZ intake. This work applied a high-throughput sequencing technology to systematically analyze changes in the fecal microbiota composition after PHZ supplementation. Principal components analysis (PCA) was firstly conducted to visualize differences in the fecal microbiota structure among the four groups (Figure 2a; PC1 and PC2 was 41.5% and 10.1%, respectively). Distinct group-based clustering patterns were observed in the score plot. Symbols representing the HFD mice were all distributed to the right quadrants while symbols representing the majority of mice of the three other groups were located to the left quadrants, suggesting drastic differences in the fecal microbiota structure between the HFD mice and those of the other three groups. Although a distinct clustering pattern was shown among the HFD+PHZ mice at the lower left quadrants, they were clustered closely with the NCD and NCD+PHZ mice (mainly distributed to the upper left quadrants on the score plot), indicating these three groups shared higher similarity in their gut microbiota structure though obvious differences still existed. These results together suggested that HFD significantly altered the gut microbiota structure; however, coadministering PHZ effectively inhibited or mitigated the drastic shift in gut microbiota. Symbols representing the NCD and NCD+PHZ mice were mainly distributed to the left upper quadrants, appearing as two closely located clusters, suggesting that a clear difference still existed between the gut microbiota structure of these two groups. Such results further confirmed that supplementing PHZ could specifically impact the gut microbiota. The results of PCA largely agreed with that of the hierarchical clustering analysis (Figure 2b), except that the sample 2–1 was not clustering with any of the groups on the PCA score plot. Interestingly, based on the PCA results, apparent differences existed in the gut microbiota structure among groups and HFD did alter the gut microbiota structure drastically, yet no significant difference was detected in the α-diversity (represented by Chao1 index) across groups (Figure 2c, *P* > .05), indicating that the different interventions did not result in significant changes in taxa richness.

To further investigate the specific changes in the bacterial communities, the relative abundance of the predominant phyla and genera was compared across four groups, especially taxa responding to PHZ supplementation (Figure 2d-p). At the phylum level, *Firmicutes* and *Bacteroidetes* were the leading phyla across all groups, occupying over 80% of the total sequences (Figure 2d). The relative abundance of *Firmicutes* significantly increased by feeding HFD (*P* < .001), and such change was evidently reversed by PHZ supplementation (*P* < .001; Figure 2e). In contrast, the phylum *Bacteroidetes* showed an opposite trend (*P* < .001; Figure 2f). Interestingly, the *Firmicutes*/*Bacteroidetes* ratio, an indicator associated with obesity reported in some studies,^37^ was significantly higher in the HFD mice compared with mice of the other three groups (*P* < .001; Figure 2g). Coadministering PHZ also showed desirable effects in reversing some of the HFD-induced alterations in the gut microbiota composition, including *Clostridia* and *Bacteroidia* at the class level (*P* < .001 in both cases; Figure 2h,i), *Desulfovibrionales* and *Bacteroidales* at the order level (*P* < .01 and *P* < .001, respectively; Figure 2j,k), *Desulfovibrion* and *Muribaculaceae* at the family level (*P* < .01 and *P* < .001, respectively; Figure 2l,m), and *Bilophila* and *GCA-900066575* at the genus level (*P* < .001 in both cases; Figure 2o,p). Moreover, PHZ supplementation significantly reduced the relative abundance of two obesity-associating genera,^38,39^ *Mucispirilu* and *Bilophila*, which were enriched by HFD (Figure 2n). Modulation of the above-mentioned taxa might together contribute to the mitigating effect of PHZ on the overall gut microbiota alterations. The overall gut microbiota modulation by PHZ supplementation could reduce the fecal LPS levels, which were significantly stimulated by HFD (*P* < .001; Figure 2q). Detailed results in the gut microbiota changes are available in the Supplementary Material Figure S1-S4.

The contents of fecal SCFAs were detected by gas chromatography-mass spectrometry (Figure 2r). The HFD group had significantly less total SCFAs compared with the NCD, NCD+PHZ, and HFD+PHZ groups (*P* < .001 in all cases), suggesting that HFD inhibited SCFAs production, which was mitigated by PHZ supplementation. Specifically, the fecal levels of acetic acid, propionic acid, and butyric acid were significantly higher in the HFD+PHZ group compared with the HFD group (Table 1; *P* < .05). However, PHZ supplementation did not change the fecal isobutyric acid, isovaleric acid, and valeric acid levels significantly (Table 1).

**Table 1.** Fecal SCFAs contents of four groups of mice (μg/mg)

Groups	Acetic acid	Propionic acid	Butyric acid	Isobutyric acid	Valeric acid	Isovaleric acid
NCD	1.10 ± 0.06^a^	0.33 ± 0.03^a^	0.22 ± 0.02^a^	0.13 ± 0.01^a^	0.04 ± 0.00^a^	0.05 ± 0.01^a^
NCD+PHZ	1.00 ± 0.08^a^	0.32 ± 0.04^ab^	0.20 ± 0.02^a^	0.14 ± 0.01^a^	0.04 ± 0.01^a^	0.04 ± 0.00^a^
HFD	0.22 ± 0.05^b^	0.16 ± 0.01 ^c^	0.07 ± 0.01^b^	0.12 ± 0.01^a^	0.03 ± 0.00^a^	0.04 ± 0.00^a^
HFD+PHZ	0.64 ± 0.05 ^c^	0.29 ± 0.04^b^	0.14 ± 0.01 ^c^	0.14 ± 0.01^a^	0.04 ± 0.00^a^	0.06 ± 0.01^a^

Data were expressed as mean±SEM. Significant differences were evaluated by one-way analysis of variance (ANOVA) and the Tukey test. Each parameter was compared across four groups, and different superscript letters indicated significant differences (*P < *0.05). NCD: normal chow diet; NCD+PHZ: normal chow diet with phlorizin; HFD: high fat diet; HFD+PHZ: high fat diet with phlorizin.

### PHZ ameliorated HFD-induced impairments in gut epithelial barrier and mucus secretion

**Figure 3.**
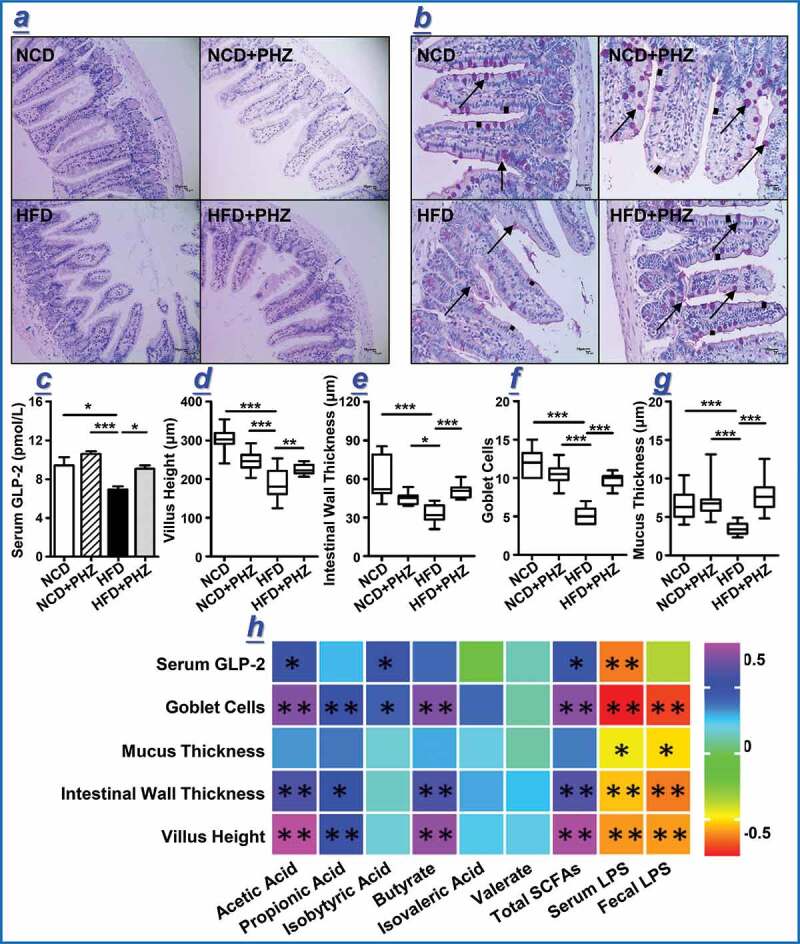
Phlorizin (PHZ) attenuated high-fat diet (HFD)-induced damage of the intestinal barrier. (a) Hematoxylin and eosin staining and (b) periodic acid-Schiff staining of intestinal tissue sections of the four groups of mice receiving normal chow diet (***NCD***), NCD with PHZ (***NCD+PHZ), HFD***, and HFD with PHZ (***HFD+PHZ***), respectively. Samples were taken at 12^th^ week. The blue marks in (a) indicate intestinal wall thickness; the arrows in (b) indicate the stained goblet cells. Various parameters indicating the integrity of intestinal barrier were analyzed at 12^th^ week, including (c) serum glucagon-like peptide-2 (GLP-2) level; (d) villus height; (e) intestinal wall thickness; (f) number of goblet cells; and (g) intestinal mucus thickness. One-way ANOVA was used to analyze statistical differences, *P < .05, **P < .01, and ***for *P* < .001. Data are expressed as mean ± standard deviation. (h) Pearson correlation analysis was performed between various fecal metabolites and indicators of gut epithelial integrity. The color scale represents the strength of correlation, ranging from 0.5 (strong positive correlation) to −0.5 (strong negative correlation)

The gut epithelial integrity is considered as the first line of defense of the gastrointestinal tract.^26^ The feeding of HFD could impair the intestinal epithelial barrier by increasing the gut LPS content;^25,40^ and our results consistently showed that HFD increased the fecal LPS level (Figure 2q). Histological analysis confirmed that HFD induced intestinal barrier damage. The hematoxylin and eosin (H&E) and alcian blue periodic acid-Schiff (AB-PAS) stainings of the gut tissues of representative mice of each group (Figure 3a,b) showed that the HFD group exhibited obvious decreases in the goblet cell quantity (shown by the arrow), mucus thickness (shown by the black short line), intestinal wall thickness (shown by the blue short line), and villus height (Figure 3a,b). The supplementation of PHZ attenuated HFD-induced damage in epithelial integrity, accompanied by an increased blood GLP-2 level (*P* < .05, Figure 3c); GLP-2 is a peptide hormone secreted by L cells that helps repair and maintains the gut barrier integrity.^41^ Quantitative histological analysis revealed significant increases in the villus height (*P* < .01; Figure 3d), intestinal wall thickness (*P* < .001; Figure 3e), number of goblet cells (deep purple stained goblet cells in Figure 3b; *P* < .001, Figure 3f), and mucus layer thickness (*P* < .001; Figure 3g) in the HFD+PHZ mice compared with the HFD mice, suggesting an overall protective effect of PHZ against HFD-induced damage of the gut epithelial barrier.

Correlation analysis was then performed to identify association between the gut microbiota/metabolites and epithelial integrity-related parameters (Table 2; Figure 3). The results of the analysis revealed that most of the key bacterial species correlated positively with the levels of serum and fecal LPS while correlated negatively with SCFAs, including acetic acid, propionic acid, butyric acid, and total SCFAs (Table 2). The relative abundance of a number of these bacterial taxa, e.g., *Eubacterium oxidoreducens* group, *Christensenellaceae R-7* group, *Rikenellaceae RC9 gut* group, *Ruminococcaceae* (Figure S3c), *Lachnospiraceae, Desulfovibrio, Helicobacter, Bilophila* (Figure S4c), and *Mucispirillum* (Figure S4f), increased significantly in the HFD mice. In the fecal samples of the HFD+PHZ mice, the relative abundance of these taxa often decreased, which was most likely resulted from PHZ supplementation. Some other species (e.g., *Phascolarctobacterium*) did not correlate with LPS but correlated negatively with SCFAs. Conversely, several key species (including *NS3a marine* group, *Tyzzerella 3*, and *ASF356*) were negatively correlated with LPS but were positively correlated with SCFAs; and their relative abundance was significantly higher in the HFD+PHZ mice compared with the HFD mice.

**Table 2.** Correlations between key gut microbial taxa and metabolites [short-chain fatty acids (SCFAs) and lipopolysaccharide (LPS)]

Taxa	Acetic acid	Propionic Acid	Isobutyric Acid	Butyrate acid	Isovaleric acid	Valerate Acid	Total SCFAs	Blood LPS	Fecal LPS
*Eubacterium oxidoreducens group*	**−.593**	**−.534**	**−.417**	**−.591**	−.349	−.272	**−.629**	.**557**	.**631**
*Ruminococcaceae NK4A214 group*	**−.405**	**−.457**	−.277	−.363	−.119	−.182	**−.439**	.332	.**531**
*Lachnospiraceae FCS020 group*	**−.430**	−.374	−.350	**−.418**	−.289	−.121	**−.458**	.278	.**551**
*Christensenellaceae R-7 group*	**−.506**	**−.466**	**−.420**	**−.539**	−.298	−.297	**−.550**	.249	.**409**
*Rikenellaceae RC9 gut group*	**−.486**	−.387	−.359	**−.480**	−.282	−.202	**−.504**	.**428**	.**592**
*Ruminococcaceae UCG-004*	.036	−.005	.019	.101	−.129	−.042	.035	−.160	.074
*Ruminococcaceae UCG-003*	**−.554**	**−.462**	−.088	**−.509**	−.122	−.266	**−.565**	.**454**	.**610**
*Ruminococcaceae UCG-009*	**−.693**	**−.591**	−.090	**−.620**	−.136	−.336	**−.702**	.**544**	.**758**
*Lachnospiraceae UCG-004*	**−.411**	**−.419**	−.355	−.397	−.403	−.258	**−.453**	.**418**	.**444**
*Lachnospiraceae UCG-008*	**−.557**	**−.533**	−.345	**−.556**	−.355	−.318	**−.602**	.**474**	.**576**
*Lachnospiraceae UCG-006*	**−.689**	**−.577**	−.248	**−.665**	**−.439**	−.290	**−.713**	.**584**	.**763**
*Phascolarctobacterium*	−.091	−.136	−.291	−.244	−.203	.119	−.137	.027	.002
*Erysipelatoclostridium*	**−.470**	−.301	−.327	**−.486**	−.310	−.099	**−.479**	.360	.**514**
*NS3a marine group*	.019	−.053	−.091	.155	.156	.133	.029	−.196	−.119
*Ruminiclostridium 5*	**−.606**	**−.508**	−.015	**−.563**	−.137	−.309	**−.614**	.**495**	.**651**
*Ruminiclostridium 9*	**−.721**	**−.623**	−.233	**−.687**	−.332	−.298	**−.747**	.**618**	.**815**
*Lachnoclostridium*	**−.614**	**−.609**	−.232	**−.568**	−.334	**−.424**	**−.655**	.**484**	.**636**
*Ruminococcus 1*	−.040	−.060	−.049	−.028	−.238	−.106	−.062	.012	.211
*GCA-900066575*	**−.568**	−.376	−.028	**−.555**	−.103	−.205	**−.556**	.**564**	.**714**
*Anaerotruncus*	**−.623**	**−.518**	−.050	**−.560**	−.094	−.304	**−.627**	.**498**	.**657**
*Oscillibacter*	**−.470**	**−.416**	−.311	**−.458**	−.404	−.165	**−.503**	.**421**	.**518**
*Acetatifactor*	**−.515**	**−.429**	.091	**−.471**	.034	−.194	**−.513**	.382	.**567**
*Harryflintia*	**−.683**	**−.617**	−.162	**−.635**	−0.28	−.308	**−.708**	.**594**	.**733**
*Tyzzerella 3*	.**467**	.375	.123	.381	−.017	.048	.**464**	.110	−.238
*ASF356*	.**552**	.262	.018	.**485**	−.043	−.139	.**507**	−.229	**−.438**
*Roseburia*	−.158	−.366	−.306	−.142	−.269	.022	−.220	.039	.222
*Blautia*	−.131	−.208	−.094	−.214	−.167	.004	−.176	.072	.129
*Alistipes*	−.040	−.013	.192	−.034	.058	.315	−.022	.162	−.081
*Desulfovibrio*	**−.541**	−.331	.066	**−.431**	−.022	−.265	**−.509**	.401	.**413**
*Helicobacter*	**−.462**	**−.428**	−.315	**−.419**	−.369	−.242	**−.492**	.**407**	.**459**
*Bilophila*	**−.492**	−.395	.098	**−.439**	.060	−.215	**−.485**	.366	.**534**
*Verruc-01*	**−.492**	**−.423**	−.397	**−.490**	−.278	−.155	**−.518**	.**421**	.**453**
*Mucispirillum*	**−.507**	**−.421**	.065	**−.447**	−.014	−.247	**−.505**	.376	.**507**
*A2*	**−.709**	**−.590**	−.367	**−.666**	−.296	−.322	**−.731**	.**582**	.**727**

Statistically significant Pearson correlations are in boldface type.

Besides, the results of correlation analysis also revealed positive correlation between SCFAs levels (especially acetic acid, propionic acid, butyric acid, and isobutyric acid) and the indicators of gut barrier integrity (including villus height, intestinal wall thickness, mucus thickness, and goblet cell quantity), as well as the blood GLP-2 level (Figure 3h). However, the indicators of gut barrier integrity was correlated negatively with the LPS level (Figure 3h).

### Transplantation of gut microbiota of HFD+PHZ mice attenuated HFD-induced damage of gut epithelial integrity and obesity

**Figure 4.**
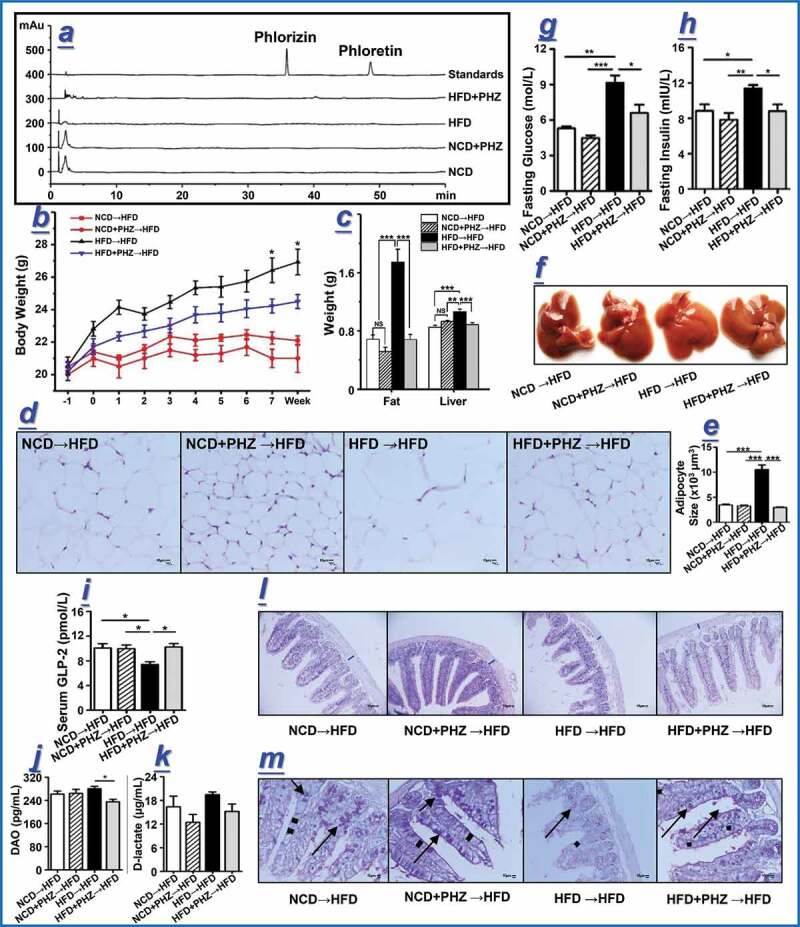
Transplantation of feces from mice fed phlorizin (PHZ) attenuated high-fat diet (HFD)-induced obesity and damage of intestinal barrier. (a) Chromatograms of PHZ and phloretin (PHT) in the feces of the four groups of donor mice fed normal chow diet (***NCD***), NCD with PHZ (***NCD+PHZ), HFD***, and HFD with PHZ (***HFD+PHZ***). The four groups of recipient mice (maintained on HFD) that underwent fecal microbiota transplantation were designated as “***NCD→HFD***”, “***NCD+PHZ→HFD***”, “***HFD→HFD***”, and “***HFD+PHZ→HFD***”, respectively. (b) Changes in body weight over eight weeks. A number of obesity- and gut epithelial barrier integrity-related parameters were recorded at eighth week, including (c) the weights of epididymis fat and liver; (d) hematoxylin and eosin (H & E) staining of epididymis fat; (e) adipocyte size of epididymis fat; (f) liver morphology; (g, h) fasting blood glucose and insulin levels; (i-k) serum levels of glucagon-like peptide-2 (GLP-2), diamine oxidase (DAO), and D-lactate; (l, m) H&E and AB-PAS staining of intestinal tissue sections. The blue marks in (l) indicate intestinal wall thickness; the arrows in (m) indicate the stained goblet cells. Data are expressed as mean ± standard deviation. One-way ANOVA was used to analyze statistical differences; NS for *P* > .05, *P < .05, **P < .01, and ***P < .001

To confirm that the attenuation of HFD-induced adverse effects was a result of the action the gut microbiota but not residues of PHZ (and its metabolites like PHT) remained in the feces of the donor mice, targeted chemical analysis was performed by using HPLC to quantify the spectrum of PHZ-related compounds in the collected feces before FMT. The two peaks, corresponding to the PHZ and PHT standards, appeared on the HPLC chromatograph of the standards but not detected in the fecal samples of the four groups of donor mice, suggesting that no PHZ or PHT was present in those samples (Figure 4a). The feces of the four groups of donor mice were transplanted daily to a new batch of acclimatized mice, which were then maintained on HFD for eight weeks, and the changes in their body weight were monitored (Figure 4b). A number of obesity- and gut epithelial barrier integrity-related parameters were recorded at eighth week, including the weights of epididymis fat and the liver (Figure 4c), H & E staining of epididymis fat (Figure 4d), adipocyte size of epididymis fat (Figure 4e), liver morphology (Figure 4f), fasting blood glucose and insulin (Figure 4g,h), serum levels of GLP-2, diamine oxidase (DAO), and D-lactate (Figure 4i-k), H&E and AB-PAS stainings of intestinal tissue sections (Figure 4l,m).

Transplantation of feces from the HFD+PHZ mice significantly attenuated HFD-induced weight gain and blood glucose increase in the recipient mice, accompanied by improvement in insulin resistance and reduction in serum LPS, suggesting that PHZ-modulated microbiota could attenuate HFD-induced MDs and that the gut microbiota was a target for the amelioration effect of PHZ (Figure 4 and **S6**). Our conclusion was also supported by a significantly higher serum GLP-2 level in the mice transplanted with feces obtained from the HFD+PHZ group than those receiving feces donated by the HFD group (*P < *.05; Figure 4i), suggesting that PHZ-modulated microbiota could stimulate GLP-2 secretion. Moreover, the HFD-induced damage of gut epithelial integrity was also attenuated after transplanting the PHZ-modulated microbiota into HFD fed mice, as evidenced by the microscopic morphology of the intestinal tissues (Figure 4l,m), as well as the significant increases in the intestinal wall thickness (Figure S6g), villus height (*P* < .01) (Figure S6h), thickeness of mucus layer (*P* < .001; Figure S6i), and number of goblet cells (*P* < .01; Figure 4m and S6j).

The gut barrier permeability and function were evaluated by the serum levels of DAO and D-lactate. The enzyme, DAO, is usually located at the upper villi of the small intestinal mucosa. D-lactate is an intestinal bacterial metabolite produced in the gut, which is not metabolizable by mammals due to the lack of a corresponding rapid enzyme system. Therefore, the serum levels of DAO and D-lactate could reflect the gut epithelial permeability and integrity.^42^ As shown in Figure 4j,k, the serum levels of DAO were 261.63 ± 10.60 pg/mL and 280.25 ± 7.96 pg/mL in mice transplanted with feces obtained from the NCD mice and the HFD mice, respectively, suggesting that transplanting the feces of the HFD mice could increase the serum level of DAO in the recipient mice. A similar increase was observed in serum D-lactate, which were 16.37 ± 2.68 μg/mL and 19.44 ± 0.68 μg/mL in mice receiving the feces from the NCD mice and the HFD mice, respectively. Such results suggested a higher gut barrier permeability in recipients transplanted with feces of the HFD mice compared with those receiving feces obtained from the NCD mice. However, the recipients transplanted with feces obtained from the HFD+PHZ mice had lower levels of serum DAO and lactate (235.08 ± 8.71 pg/mL and 15.18 ± 1.89 μg/mL, respectively) compared with mice receiving feces of the HFD mice, suggesting that transplanting PHZ-modulated microbiota could attenuate HFD-induced damage of the gut barrier.

## Discussion

Our results confirmed that supplementing PHZ significantly inhibited HFD-induced body weight gain and fat accumulation in mice, which was accompanied by a decrease in the serum LPS level and improvement in insulin resistance. However, the low concentration of intact PHZ detected in the blood circulation made it hard to fully explain its pharmacological mechanism. Indeed, after oral intake, a large portion of the ingested PHZ would pass through the gastrointestinal tract. Thus, this study tested the hypothesis that the gut epithelial barrier and the gut microbiota were novel therapeutic targets for the pharmacological action of PHZ, which is a continuation of our previous research in *db/db* mice indicated by non-sequencing-based method, the denaturing gradient gel electrophoresis (DGGE).^36^ By using a HFD-induced obese mice model and FMT, this study has provided evidence that the ‘gut microbiota-barrier axis’ was indeed an alternative target for the anti-obesity effect of PHZ.

Our study first confirmed that the gut microbiota was a potential target for PHZ to improve MDs, partly evidenced by the reduced gut LPS and increased intestinal SCFAs levels. As two kinds of gut microbiota-originated metabolites, LPS and SCFAs are widely regarded as classical indicators of the state of the gut system and the function of the gut microbiota.^43^ Our results observed that PHZ supplementation significantly decreased the levels of some LPS-producing and obesity-related bacterial genera,^44–47^ such as *Desulfovibrio, Ruminiclostridium, Anaerotrucus*, and *Oscillibacter*, in HFD mice, leading to the decline in the overall fecal LPS level.

Correlation analysis revealed that the spectrum of bacteria that was induced by HFD correlated positively with LPS but negatively with SCFAs, and PHZ supplementation attenuated the increase in LPS and decrease in SCFAs. Bacterial genera, such as *Eubacterium oxidoreducens* group, *Christensenellaceae R-7* group, *Rikenellaceae RC9 gut* group, *Ruminococcaceae, Lachnospiraceae, Desulfovibrio, Helicobacter, Bilophila*, and *Mucispirillum*, were found to increase in mice receiving HFD, but the relative abundance of these genera decreased in the fecal samples of the HFD+PHZ mice. On the other hand, coadministering PHZ significantly promoted the growth of taxa that correlated positively with SCFAs but negatively with LPS (e.g., *NS3a marine* group, *Tyzzerella 3*, and *ASF356*).

Lipopolysaccharide is considered as one of the main triggers for chronic inflammation and even MDs;^20^ it can destroy and penetrate the intestinal barrier,^48^ particularly after binding to chylomicrons.^17^ Impairment of the intestinal barrier function has been associated with various intestinal and systemic diseases.^25,49,50^ Therefore, we speculated that the significant decrease in LPS-producing bacteria resulted from the PHZ treatment helped reduce the fecal LPS and subsequent damage to the physical gut barrier, limiting the leakage of LPS to the bloodstream.

Another significant group of microbial metabolites involved in the anti-obseity effect of PHZ was SCFAs; SCFAs play critical roles in regulating host physiological function and intestinal homeostasis,^51^ particularly enhancing the gut barrier integrity.^26,34^ Our study found that feeding PHZ to HFD-induced obese mice significantly attenuated the reduction in fecal SCFAs in these mice, especially acetic acid, propionic acid, and butyric acid. In fact, SCFAs have been reported to directly involve in hormone secretion and promote the differentiation and activity of L cells,^34^ which are distributed throughout the epithelial layer of the gut wall.^26^ Some of the hormones that are regulated by SCFAs include GLP-1 and GLP-2; GLP-1 enhances glucose-dependent insulin secretion,^52^ while GLP-2 is known to participate in repairing and maintaining the gut barrier integrity.^26^ Therefore, another possible mechanism of the anti-obesity action of PHZ was likely via stimulating the SCFAs-producing bacteria and subsequently increasing the colonic SCFAs contents, which further regulated hormone secretion, leading to gut barrier restoration and insulin resistance reversal.

**Figure 5.**
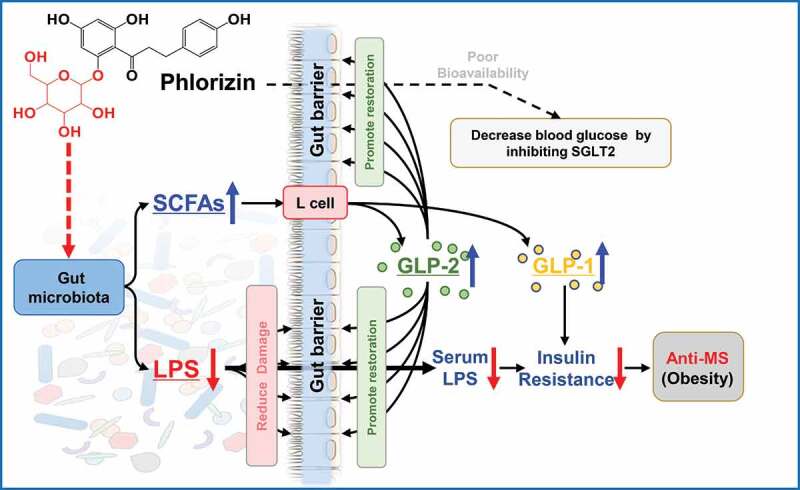
Schematic diagram showing the possible mechanisms of anti-metabolic disorders (MDs) effects of phlorizin (PHZ). Other than the primary target of sodium-glucose co-transporters-2 (SGLT2), the ‘gut microbiota-barrier axis’, including the gut microbiota and the gut barrier, served as alternative targets for the beneficial action of PHZ. GLP: glucagon-like peptide; LPS: lipopolysaccharide; SCFAs: short-chain fatty acids

To further test our hypothesis that the gut barrier and the gut microbiota were alternative targets for the anti-obesity action of PHZ, an FMT experiment was performed by transplanting the feces of mice fed NCD or HFD with/without PHZ coadministration. Before the FMT, HPLC analysis was performed to confirm that no PHZ and related compounds like PHT were present in the feces samples to be transplanted to the recipient mice. Indeed, the ingested PHZ would enter the gastrointestinal tract, and it might thus act via a two-step mechanism: 1. a large portion of unabsorbed PHZ reached the gastrointestinal tract and some of which was metabolized to PHT; 2. these compounds modulated the gut microbiota locally in a similar manner as prebiotics. As a result, LPS production was inhibited, and LPS-induced damage to the gut epithelial barrier was reduced. Meanwhile, the SCFAs levels increased and in turn stimulated GLP-2 secretion. The GLP-2 subsequently helped repair the damaged physical barrier of the gastrointestinal tract and effectively prevent leakage of LPS into the systemic circulation and endotoxemia. Despite the low bioavailability of PHZ and absorption to the bloodstream to act via competitive inhibition of the SGLTs, our findings supported that the combined action of some effector molecules, e.g., SCFAs and GLP-2, together contributed to the amelioration of HFD-induced MDs, as indicated by an improved insulin resistance (Figure 5).

A growing quantity of evidence has shown that plant-sourced phenols (including flavonoids, phenolic acids, hydroxyphenols, and catechins) are effective in preventing and treating MDs by improving the colon antioxidant and anti-inflammatory status, thus enhancing the epithelial barrier function and/or modulating the gut microbiota.^53^ Our FMT experiment showed convincingly that the gut epithelial barrier and the gut microbiota were alternative targets for the therapeutic action of PHZ; however, it is important to note that more efforts should focus on characterizing both the microbiota-derived and phenols-derived metabolites in the feces of the donors for biosafety when applying FMT.^54^ Previous metabolomic analysis has found that the functional effectors of the beneficial function were often not the phenols but their metabolites.^55^ For example, most natural compounds in the form of glycosides would need to be activated by a first hydrolytic step to the more bioactive form, aglycones.^56^ Similarly, PHZ would usually be acidolyzed and/or enzymatically hydrolyzed to PHT after entering the gastrointestinal tract;^57^ thus, it was unclear if the anti-obesity and gut microbiota modulatory effects were due to the original form of PHZ and/or its aglycone, PHT. Particularly, our preliminary study found that PHT exhibited a much stronger *in vitro* antibacterial effect than PHZ (data not shown). Moreover, it was likely that the unabsorbed PHZ/PHT entered the digestive tract and the gut microbiota were mutually interactive. On one hand, the PHZ and PHT could modulate the gut microbiota composition, and on the other hand, PHZ and PHT might serve as substrates to be metabolized by the gut microbes and in turn release other metabolites to stimulate downstream metabolism. Finally, to confirm the role of the gut microbiota and that it was the target of action of PHZ in the anti-obesity effect, it would be critical to ensure that no PHZ and PHT residues were present in the feces to be transplanted, which could sometimes be excreted and detected in the fecal samples when a high dose of PHZ was administered. It would be equally important to ensure that no other glycosides and/or phenols of low bioavailability like PHZ were present in the feces to be transplanted to the recipient mice. Otherwise, it might be hard to validate the gut microbiota as an alternative target of PHZ or to decipher the role of PHZ in the anti-obesity action.

In addition, since the hydrolysis or metabolization of PHZ was not instantaneous, it would be necessary to analyze the digestion kinetics of PHZ. It would also be of interest to investigate whether PHZ and/or its metabolites could directly stimulate hormone secretion that contributes to the anti-MDs effect, which may further elucidate the molecular mechanism of PHZ as a phytonutrient. The current findings have provided an explanation for the high efficacy of PHZ despite its low bioavailability, and PHZ holds great potential to be developed as a functional food ingredient.

## Materials and methods

### Materials and reagents

The PHZ (≥98%) was purchased from Changsha Zhongren Biotechnology Co., Ltd. (Changsha, China), and it was dissolved in 0.9% of sterile saline before use. The PHZ standard was bought from Chengdu Herbpurify Co., Ltd. (No. Y-029-121123; Chengdu, Sichuan, China). Both acetonitrile and methanol (HPLC grade) were obtained from Tedia Co., Inc. (Fairfield, Ohio, USA). A Milli-Q water purification system (Millipore, Bedford, Massachusetts, USA) was used to produce deionized water (18.25 MΩ). All other chemicals were at least of analytical grade.

### Animal studies

The Animal Ethics Committee of Sichuan University (No. KY160049) approved all the animal experiments. Male C57BL/6 J mice aged six weeks were bought from Chengdu Dossy Experimental Animals Co., Ltd. (Sichuan, China) and were acclimatized for one week under a controlled environment (temperature range of 20–22°C; relative humidity between 40%-60%; under a 12 h light/dark cycle by switching on lights at 8:00 AM, lights off at 8:00 PM). Food and water were available *ad libitum* during acclimatization.

After the acclimatization, the mice were assigned to four groups (n = 10 per group) in a feeding experiment: (1) *NCD* group (fed NCD, 10% of calories derived from fat; Supplementary Material Table S1); (2) *NCD+PHZ* group (fed NCD and 80 mg/kg of PHZ intragastrically); (3) *HFD* group (fed HFD, 60% of calories derived from fat; Supplementary Material Table S1); and (4) ***HFD+PHZ*** group (fed HFD and 80 mg/kg of PHZ intragastrically). All interventions lasted 12 weeks, and the body weight and food intake of mice were measured each week. The feces of the four groups of mice (as donor mice) were collected every day during the first four weeks of the feeding experiment for FMT and for further analysis. The fecal samples were collected and stored at −80°C until use.

**Figure 6.**
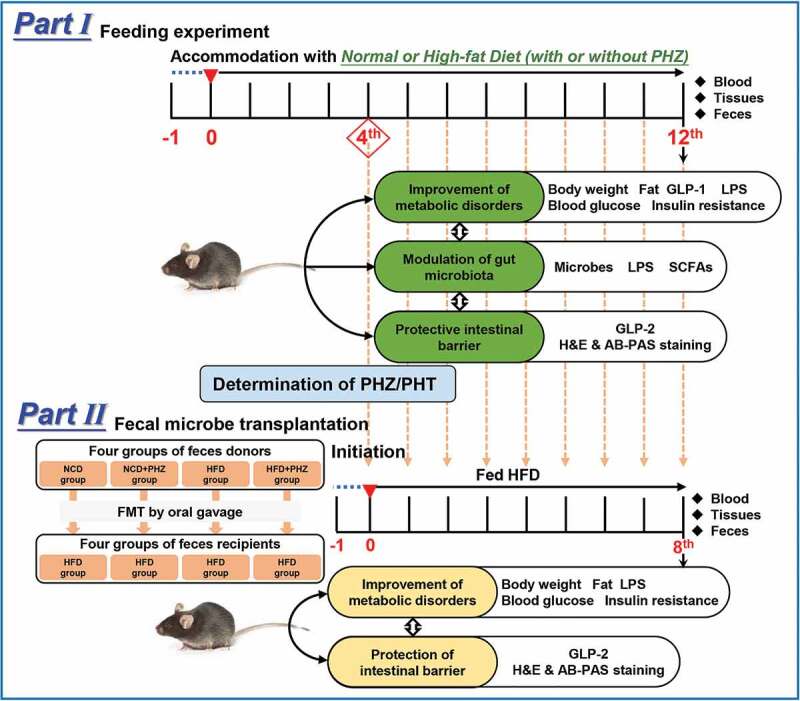
Experimental design. PHZ: phlorizin; PHT: phloretin; GLP: glucagon-like peptide; LPS: lipopolysaccharide; SCFAs: short-chain fatty acids; H&E: hematoxylin and eosin; AB-PAS: periodic acid-Schiff; NCD: normal chow diet; HFD: high-fat diet. FMT: fecal microbiota transplantation

At week 12, OGTT and ITT were performed using 2.0 g/kg glucose and 0.75 U/kg insulin, respectively, according to the methods described by Zhou et al.^58^ After fasting for 12 h, all mice were sacrificed. Serum samples were collected by centrifuging the collected blood at 1700 rpm for 10 min, and the supernatants were stored at −80°C. Tissues and organs, such as intestine, liver, and epididymis fat, were also stored at −80°C. Formalin solution (10%, buffered) was used to fix parts of the intestine and epididymis fat collected from the mice. The experimental design is presented in Figure 6.

### Fecal microbiota transplantation

Before FMT, all recipient mice (a total of 24 mice, n = 6 per group) were acclimatized for one week as described above. After the acclimatization, the mice were fed a HFD throughout the FMT experiment. For FMT, the fecal samples daily collected from each group of donor mice from the fourth week of the feeding experiment were homogenized and suspended in 0.9% of sterile saline (200 mg/2 mL), followed by centrifugation at 800 rpm for 3 min. Each recipient mouse received 100 μL of respective bacterial suspension intragastrically, i.e., ***NCD→HFD*** group, ***NCD+PHZ→HFD*** group, ***HFD→HFD*** group, and ***HFD+PHZ→HFD*** group, correspondingly. The FMT was performed daily from the fourth week (Figure 6). The body weight and the food intake of the mice were recorded weekly. The fecal samples of each group of recipient mice were separately collected daily and stored at −80°C for further analysis. At eighth week of the FMT experiment, OGTT and ITT were performed according to the methods described by Zhou et al,^58^ and samples of blood, tissues, and organs were also collected as described above after sacrificing the mice (Figure 6).

### Biochemical analysis

The fecal LPS, serum LPS, GLP-1, GLP-2, DAO, D-lactic acid, and insulin were quantified by commercially available enzyme-linked immunosorbent assay (ELISA) kits (Yinggong, Inc., Shanghai, China) according to the manufacturer’s instructions. Before the measurement of fecal LPS by ELISA, the LPS was extracted according to methods described by Han et al.^59^

### Histopathological analysis

The epididymis fat and ileum section (3 to 5 cm above the cecum) of each mouse were collected for histopathological analysis. The H&E and AB-PAS stainings were used to stain the tissue sections according the methods described by Zhang et al.^60^ A trinocular compound microscope with digital camera (BA410 Digital, Motic Group Co., Ltd.) was used to measure the size of adipocyte cell, small intestinal villus height, intestinal wall thickness, goblet cells quantity, and intestinal mucus thickness.

### Determination of PHZ and PHT in feces before FMT

Prior to FMT, the fecal samples collected from the donor mice were extracted with methanol (*m:v*, 1:4) by ultrasonic extraction (250 W, 25°C for 20 min). After centrifugation (3000 rpm for 10 min) and filtration, the supernatants were collected. An established HPLC method was used to quantify the contents of PHZ and its aglycone, PHT, in the collected supernatants.^7^

### Gut microbiota analysis

The methods for analyzing the gut microbiota diversity and taxonomic profiles of the four groups of donor mice were described in detail in the **Supplementary Methods**.

### Gas chromatography-mass spectrometry analysis

Quantification of fecal SCFAs (including acetic acid, propionic acid, butyric acid, isbutyric acid, valeric acid, and isovaleric acid) was conducted by previously described methods,^61^ with slight modifications. Briefly, the fecal samples were suspended in and extracted with 25% methanol (*m:v*, 1:2.5) at 4°C for 20 min. The extracted samples were centrifuged (3400 rpm, 10 min) and filtered (membranes of 0.22 μm pore size) before analysis by using a PerkinElmer Gas Chromatography-Mass Spectrometer (Shelton, Connecticut 0648 USA) with a polar DB-FFAP capillary column (30 m × 0.25 mm i.d., film thickness of 0.25 μm). For the chromatographic separation, helium with a purity of 99.999% was utilized as carrier gas at a constant flow rate of 1 mL/min; hydrogen with a purity of 99.999% was utilized as auxiliary gas with the flame ionization detection (FID) temperature of 250°C, the injection port temperature of 250°C, and the shunt ratio of 10:1. For the analysis, 1 μL of sample was detected by the following temperature gradient: temperature was set to 100°C initially, then increased to 180°C at 5°C per min and maintained for 2 minu, and increased again to 250°C at 20°C per min and maintained for more than 5 min. The contents of SCFAs were quantified by using an external standard method with a standard mixture of SCFAs (Supplementary Material Table S2 and Figure S6).

### Statistical analysis

The SPSS software (version 20.0, Chicago, Illinois, USA), GraphPad Prism software (version 5.01, San Diego, California, USA), and Origin Pro 9 software (Origin Lab Corporation, Wellesley Hills, Massachusetts, USA) were used to perform statistical analysis. One-way analysis of variance (ANOVA) and Tukey test were used to determine significant differences. All data were expressed as mean ± standard deviation. A *P* value < .05 was considered statistically significant.

## Supplementary Material

Supplemental Material

Supplemental Material
