# Supplementary material for: Phlorizin ameliorates obesity-associated endotoxemia and insulin resistance in high-fat diet-fed mice by targeting the gut microbiota and intestinal barrier integrity
Source: Gut Microbes. 2020 Nov 23;12(1):1842990. doi: 10.1080/19490976.2020.1842990 (PMC7714487; doi:10.1080/19490976.2020.1842990)
Supplement: Supplemental Material [file KGMI_A_1842990_SM2404.docx]

**Supplementary Method for Gut Microbiota Analysis**

**1. Amplification of Target DNA Fragment**

**1.1 Extraction of Genomic DNA**

Total genomic DNA from samples was extracted using DNeasy PowerSoil Kit (QIAGEN) according to the descriptions. DNA concentration and quality were monitored by a NanoDrop Spectrophotometer, which was diluted to 10 ng/μL with sterile ultrapure water and stored at -80°C for downstream application.

**1.2 Amplicon Generation by Polymerase Chain Reaction (PCR)**

Primers for V4 regions of 16S rRNA: ^[^^[[1]](#endnote-1)]^

515F-(5’-GTGCCAGCMGCCGCGGTAA-3’)

806R-(5’-GGACTACHVGGGTWTCTAAT-3’)

16S rRNA genes were amplified using the specific primer with 12 nt unique barcode. The PCR mixture (25 μL) consisted of 1x PCR buffer, 1.5 mM of MgCl_2_, 0.4 μM of each deoxynucleoside triphosphate, 1.0 μM of each primer, 0.5 U of KOD-Plus-Neo (TOYOBO) and 10 ng of template DNA. The PCR amplification program consisted of initial denaturation at 94℃ for 1 min, followed by 30 cycles (denaturation at 94 ℃ for 20 s, annealing at 54 ℃ for 30 s, and elongation at 72 ℃ for 30 s), and a final extension at 72 ℃ for 5 min. Three replicates of PCR reactions for each sample were combined together.

**1.3 Quantification and Qualification of PCR Products**

PCR products mixed with 1/6 volume of 6x loading buffer were loaded on 2% agarose gel for detection. Samples with bright main strip between 200-400bp were chosen for further experiments.

**1.4 Purification and Mixing of PCR Products**

The electrophoresis band was purified by OMEGA Gel Extraction Kit (Omega Bio-Tek, USA). DNA was quantified by Qubit@ 2.0 Fluorometer (Thermo Scientific). PCR products from different samples were pooled with equal molar amount.

**1.5 Library Preparation and Sequencing**

Sequencing libraries were generated by TruSeq DNA PCR-Free Sample Prep Kit following manufacturer’s recommendations and index codes were added. The library quality was assessed on the Qubit@ 2.0 Fluorometer (Thermo Scientific) and Agilent Bioanalyzer 2100 system. At last, the library was applied to paired-end sequencing (2×250 bp) with an Illumina Hiseq apparatus at Rhonin Biosciences Co., Ltd.

**2. Sequencing Data Analysis**

**2.1 Paired-end Reads Assembly**

The sequences were analyzed according to Usearch (http://drive5.com/uparse/) and QIIME5 pipeline. Paired-end reads from the original DNA fragments were merged BY FLASH ^[^^[[2]](#endnote-2)]^. Then sequences were assigned to each sample according to the unique barcode.

**2.2 OTU Clustering and Taxonomy Assignment**

Relatively stringent quality controls were adopted. Firstly, low quality reads (length < 200 bp, more than two ambiguous base ‘N’, or average base quality score < 30) were filtered, and sequences where quality scores decay (score < 11) were truncated by Trimmomatic ^[^^[[3]](#endnote-3)]^ and Usearch. After finding duplicated sequences, all the singletons, which may be bad amplicon (http://www.drive5.com/usearch/manual/singletons.html) and led to overestimation of diversity were discarded. Sequences were clustered into operational taxonomic units (OTUs) at 97% identity threshold bt UPARSE algorithms ^[^^[[4]](#endnote-4)]^. Representative sequences were then picked and potential chimeras were removed by Uchime algorithm ^[^^[[5]](#endnote-5)]^. Taxonomy were assigned by the Silva database ^[^^[[6]](#endnote-6)]^ and uclust classifier in QIIME.

**2.3 Statistical analysis**

In case of the influences of sequencing depth on community diversity, the OTU table was rarified to make all samples holding the same sequence number. All data analysis were performed by R ^[^^[[7]](#endnote-7)]^ or Python (https://www.python.org/). Weighted and Unweighted Unifrac distances were calculated in GUniFrac ^[^^[[8]](#endnote-8)]^. Alpha and beta diversity metrics were calculated in Vegan. Rarefaction curves were generated based on these three metrics. Kruskal-Wallis rank sum test was performed to show the significance of the difference on alpha diversity and taxa among different groups. Principal component analysis (PCA) was applied to reduce the dimensions of original community data. Differential species analysis: the stamp-bio software was applied to screen the species with differences among different levels. Significant differences were evaluated by one way analysis of variance (ANOVA) and the Tukey test. A value of p < 0.05 was considered as statistically significant. All data were expressed as mean ± standard error of mean.

**Reference**

1. [] Caporaso, J. G. et al. Global patterns of 16S rRNA diversity at a depth of millions of sequences per sample. Proc Natl Acad Sci U S A 108 Suppl 1, 4516–22 (2011). [↑](#endnote-ref-1)
2. [] Magoč, T. & Salzberg, S. L. FLASH: Fast length adjustment of short reads to improve genome assemblies. Bioinformatics 27, 2957–2963 (2011). [↑](#endnote-ref-2)
3. [] Bolger, A. M., Lohse, M. & Usadel, B. Trimmomatic: A flexible trimmer for illumina sequence data. Bioinformatics btu170 (2014). [↑](#endnote-ref-3)
4. [] Edgar, R. C. UPARSE: Highly accurate otu sequences from microbial amplicon reads. Nat Methods 10, 996–8 (2013). [↑](#endnote-ref-4)
5. [] Edgar, R. C., Haas, B. J., Clemente, J. C., Quince, C. & Knight, R. UCHIME improves sensitivity and speed of chimera detection. Bioinformatics 27, 2194–200 (2011). [↑](#endnote-ref-5)
6. [] Quast, C. et al. The silva ribosomal rna gene database project: Improved data processing and web-based tools. Nucleic acids research gks1219 (2012). [↑](#endnote-ref-6)
7. [] R Core Team. R: A language and environment for statistical computing. (R Foundation for Statistical Computing, 2016). [↑](#endnote-ref-7)
8. [] Chen, J. GUniFrac: Generalized unifrac distances. (2012). [↑](#endnote-ref-8)
