# Supplementary material for: Phlorizin ameliorates obesity-associated endotoxemia and insulin resistance in high-fat diet-fed mice by targeting the gut microbiota and intestinal barrier integrity
Source: Gut Microbes. 2020 Nov 23;12(1):1842990. doi: 10.1080/19490976.2020.1842990 (PMC7714487; doi:10.1080/19490976.2020.1842990)
Supplement: Supplemental Material [file KGMI_A_1842990_SM2395.docx]

**Supplementary Materials**

**Table S1 Ingredients of NCD and HFD**

**Table S2 Standard curves of SCFAs**

**Figure S1 Gut microbiota composition at Class level among 4 groups**

**Figure S2 Gut microbiota composition at Order level among 4 groups**

**Figure S3 Gut microbiota composition at Family level among 4 groups**

**Figure S4 Gut microbiota composition at Genus level among 4 groups**

**Figure S5 PHZ-modulated microbiota inhibited the development of obesity-related symptoms and ameliorated intestinal barrier damage induced by HFD.**

**Figure S6 GC-MS Chromatogram of SCFAs among 4 groups**

# Table S1 Ingredients of NCD and HFD

|  | **Normal chow diet** | | **High fat diet** | | |
| --- | --- | --- | --- | --- | --- |
| **Serial number** | **D12450B** | | **D12492** | | |
| **Macronutrient** | **gm%** | **Kcal%** | | **gm%** | **Kcal%** |
| Protein | 19.2 | 20.0 | | 26.2 | 20.0 |
| Carbohydrate | 67.3 | 70.0 | | 26.3 | 20.1 |
| Fat | 4.3 | 10.0 | | 34.9 | 59.9 |
| Total |  | 100.0 | |  | 100.0 |
| Kcal/gm* | 3.85 |  | | 5.24 |  |
| **Ingredient** | **gm** | **Kcal** | | **gm** | **Kcal** |
| Casein, 80 mesh meshes | 200 | 800 | | 200 | 800 |
| DL-Methionine | 3 | 12 | | 3 | 12 |
| Corn starch | 315 | 1260 | | 0 | 0 |
| Maltodextrin 10 | 35 | 140 | | 125 | 500 |
| Sucrose | 350 | 1400 | | 68.8 | 275.2 |
| Cellulose BW20 | 50 | 0 | | 50 | 0 |
| Soybean oil | 25 | 225 | | 25 | 225 |
| Lard | 20 | 180 | | 245 | 2205 |
| Complex mineral | 10 | 0 | | 10 | 0 |
| Dicalcium phosphate | 13 | 0 | | 13 | 0 |
| Calcium carbonate | 5.5 | 0 | | 5.5 | 0 |
| Potassium citrate, 1 H2O | 16.5 | 0 | | 16.5 | 0 |
| Multivitamin V10001 | 10 | 40 | | 10 | 40 |
| Choline Bitartrate | 2 | 0 | | 2 | 0 |
| FD&C Yellow Dye #5 Pigment | 0.05 | 0 | | 0 | 0 |
| FD&C Red Dye #40 Pigment | 0 | 0 | | 0 | 0 |
| FD&C Blue Dye #1 Pigment | 0 | 0 | | 0.05 | 0 |
| **Total** | **1055.05** | **4057** | | **773.85** | **4057** |
| Nutrient to calorie ratio (Kcal/gm): used to measure the amount of a specific nutrient (or group of nutrients) in a diet per calorie | | | | | |

# Table S2 Standard curves of SCFAs

| **SCFAs** | **Standard curves** | **R^2^** | **X range** |
| --- | --- | --- | --- |
| Acetic acid | Y=128.58X-21.538 | 0.9983 | 0.165~5.283 |
| Propionic acid | Y=394.75X-21.722 | 0.9964 | 0.062~1.989 |
| Butyric acid | Y=664.23X-16.058 | 0.9988 | 0.031~0.978 |
| Valeric acid | Y=841.15X-10.715 | 0.9986 | 0.015~0.483 |
| Isobutyric acid | Y=701.11X-13.428 | 0.9991 | 0.015~0.473 |
| Isovaleric acid | Y=861.19X-11.762 | 0.9993 | 0.014~0.451 |

# Figure S1 Gut microbiota composition at Class level among 4 groups

**
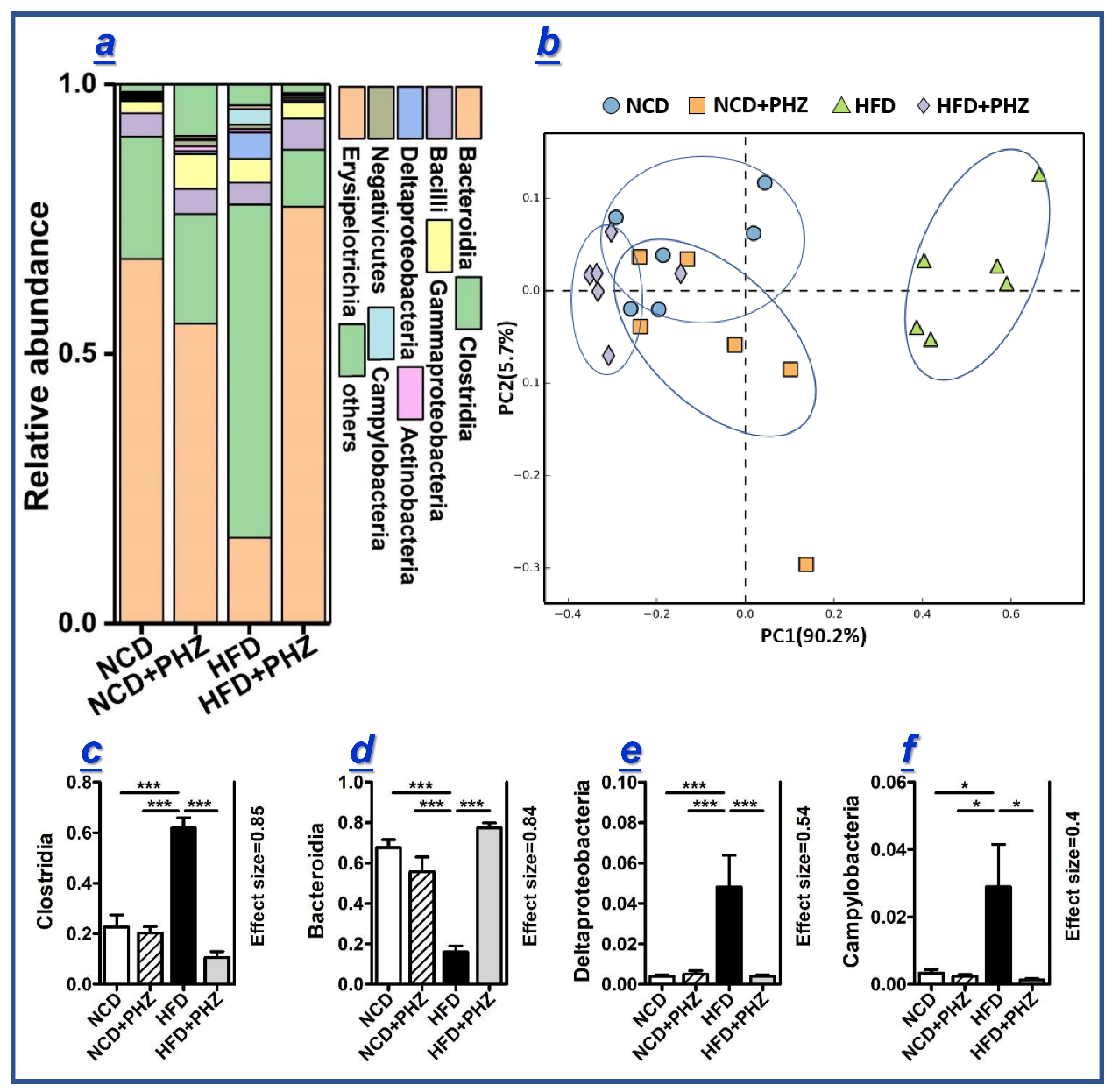
**

Data were expressed as Mean ± SEM. Differences were assessed by one-way ANOVA and denoted as follows: NS for P>0.05, * for P<0.05, ** for P<0.01 and *** for P<0.001.

# Figure S2 Gut microbiota composition at Order level among 4 groups

**
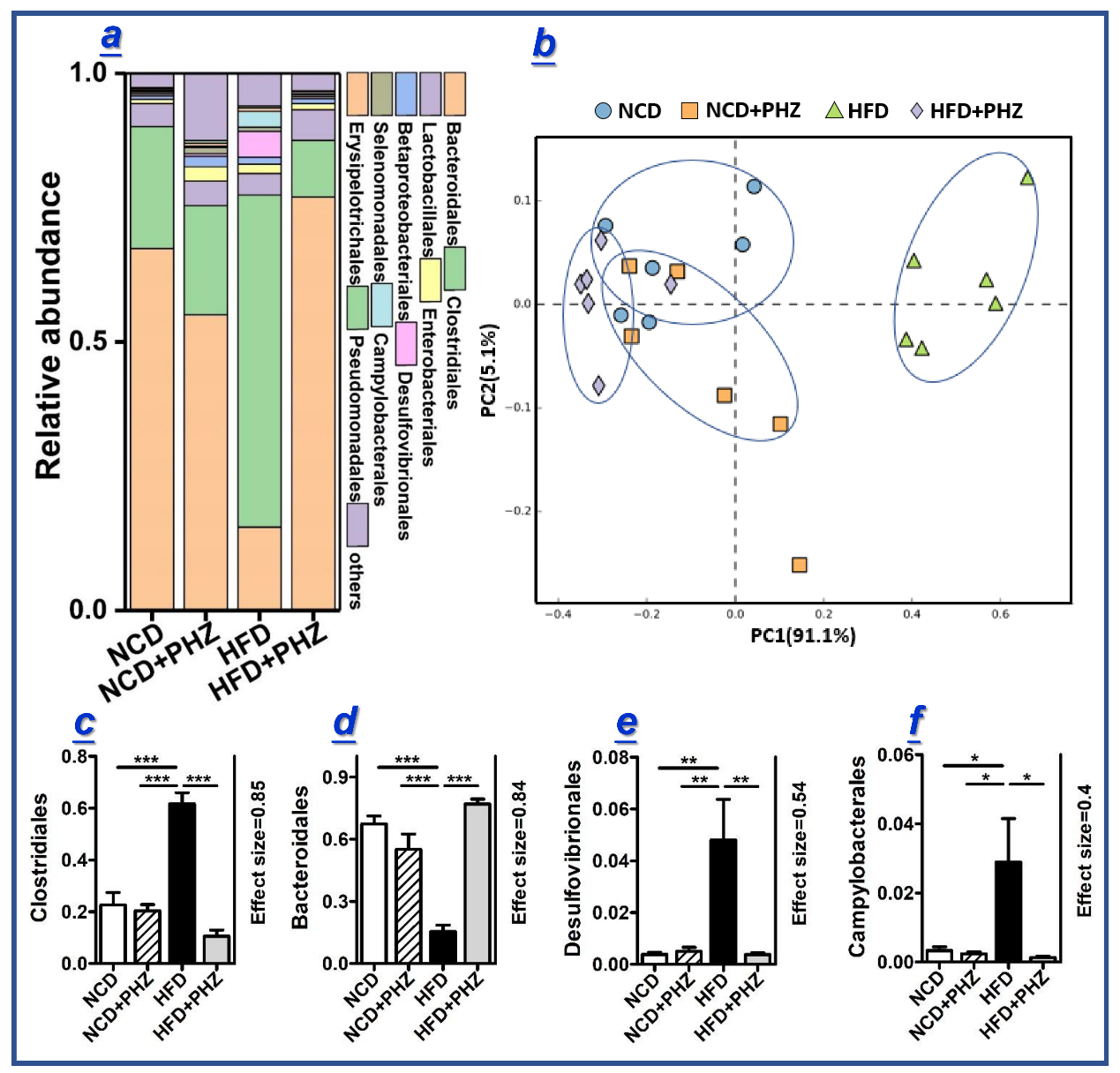
**

Data were expressed as Mean ± SEM. Differences were assessed by one-way ANOVA and denoted as follows: NS for P>0.05, * for P<0.05, ** for P<0.01 and *** for P<0.001.

# Figure S3 Gut microbiota composition at Family level among 4 groups

**
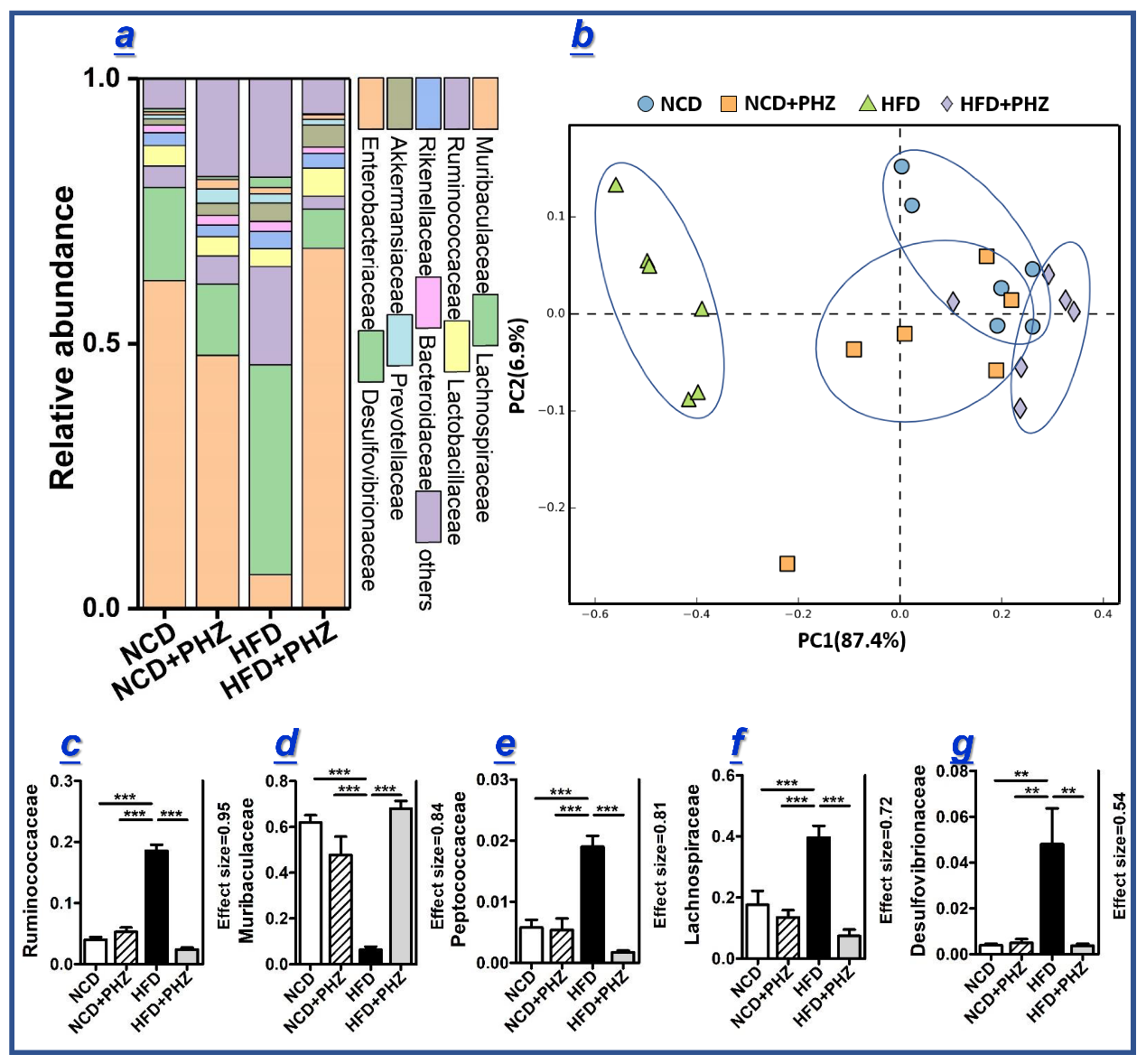
**

Data were expressed as Mean ± SEM. Differences were assessed by one-way ANOVA and denoted as follows: NS for P>0.05, * for P<0.05, ** for P<0.01 and *** for P<0.001.

# Figure S4 Gut microbiota composition at Genus level among 4 groups

**
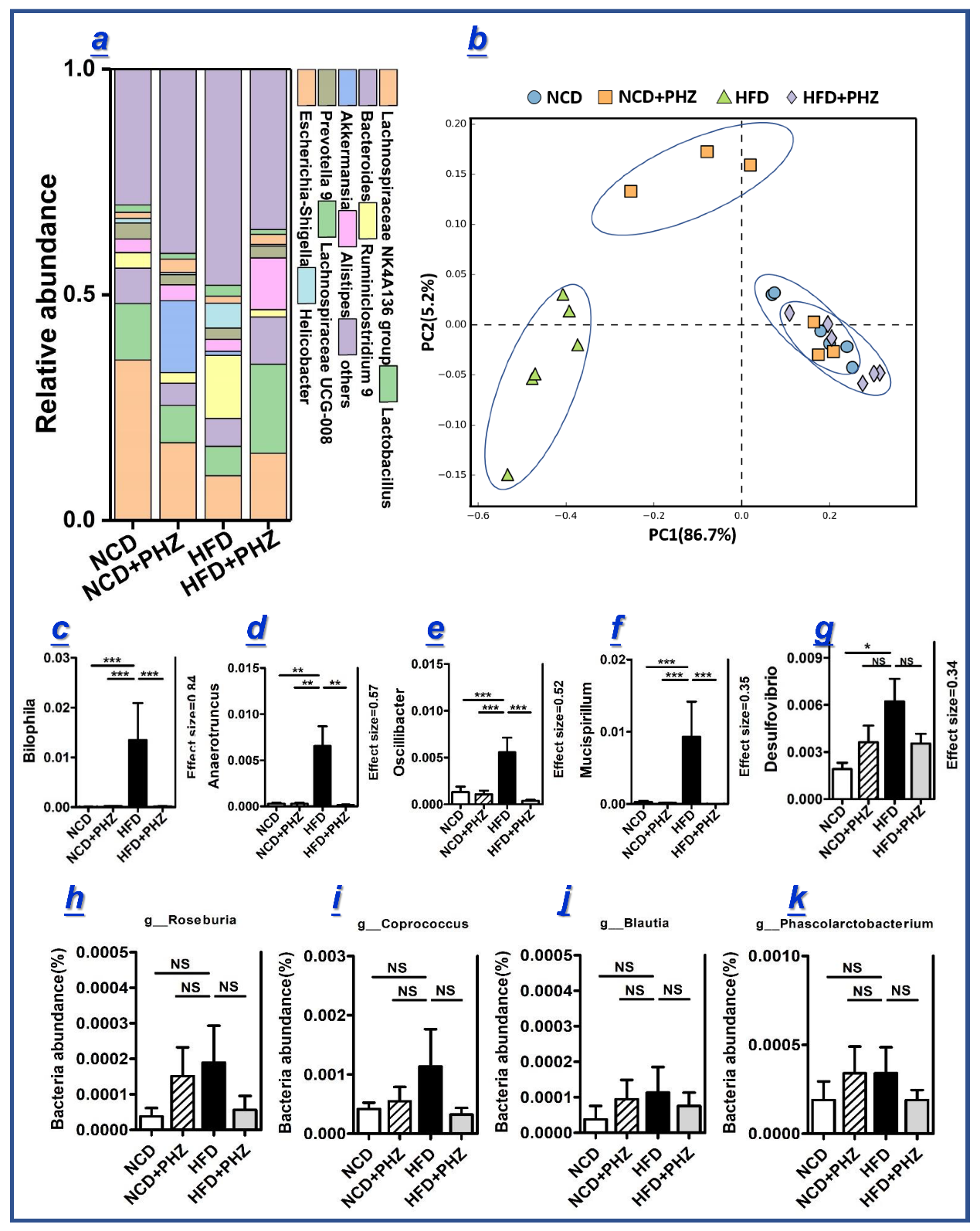
**

Data were expressed as Mean ± SEM. Differences were assessed by one-way ANOVA and denoted as follows: NS for P>0.05, * for P<0.05, ** for P<0.01 and *** for P<0.001.

# Figure S5 PHZ-modulated microbiota inhibited the development of obesity-related symptoms and ameliorated intestinal barrier damage induced by HFD.


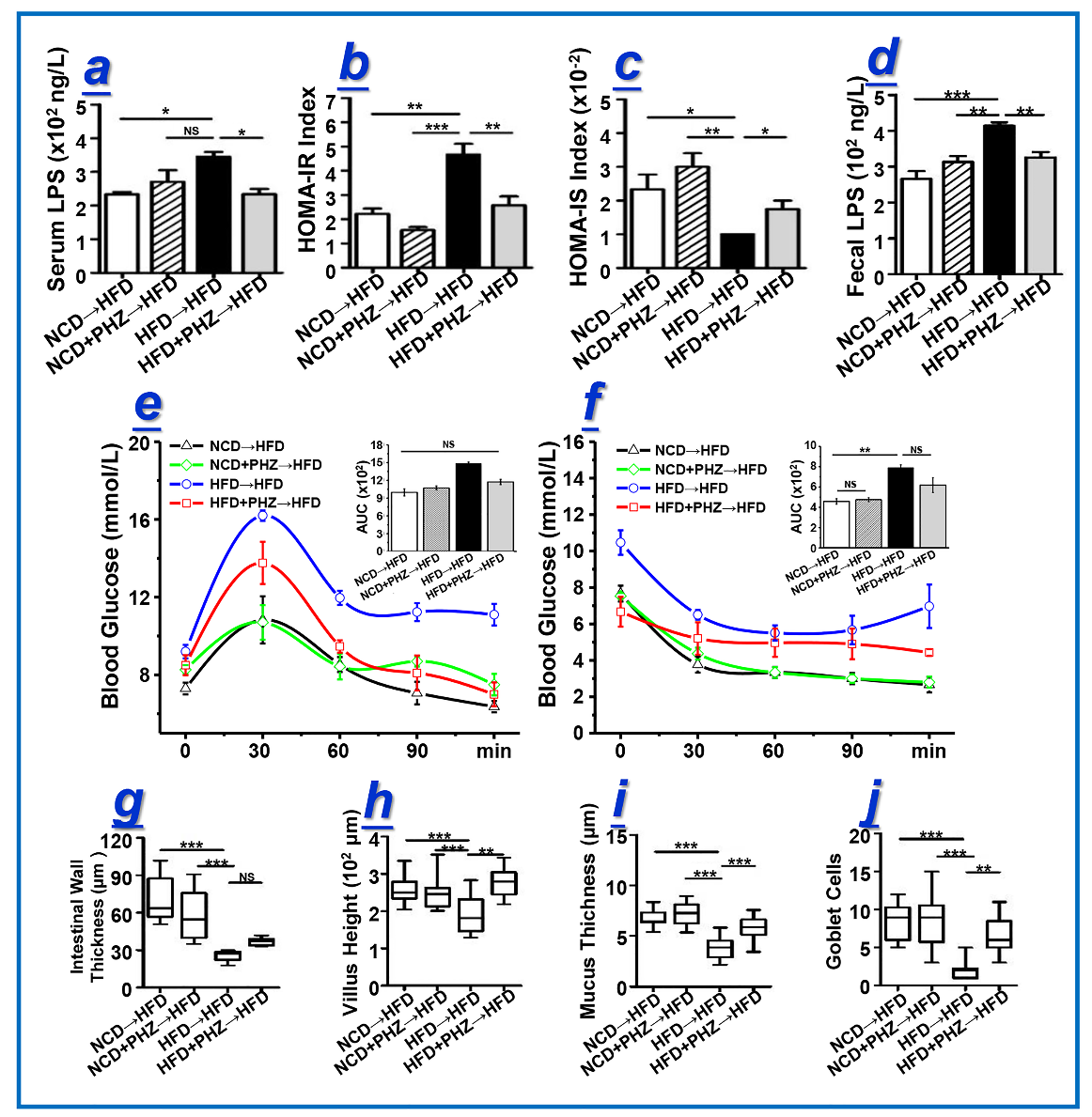


# Figure S6 GC-MS Chromatogram of SCFAs among samples


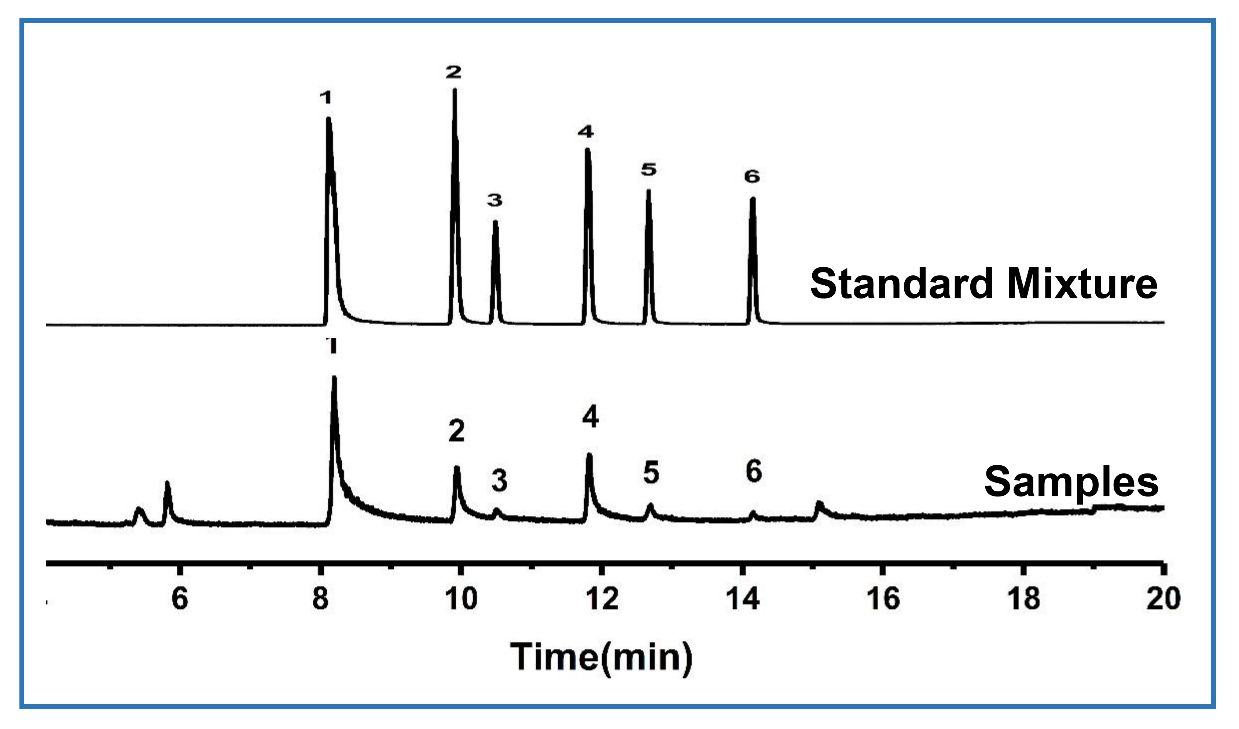


1-Acetic acid; 2-Propionic acid; 3-Isobutyric acid; 4-Butyric acid; 5-Isovalic acid; 6-Valeric acid
